# Design and experimental research of air-assisted nozzle for pesticide application in orchard

**DOI:** 10.3389/fpls.2024.1405530

**Published:** 2024-07-09

**Authors:** Mingxiong Ou, Jiayao Zhang, Wentao Du, Minmin Wu, Tianyu Gao, Weidong Jia, Xiang Dong, Tie Zhang, Suming Ding

**Affiliations:** ^1^ School of Agricultural Engineering, Jiangsu University, Zhenjiang, China; ^2^ Tillage and Pesticide Application Research Center, Chinese Academy of Agriculture Mechanization Sciences Group Co., Ltd., Beijing, China; ^3^ Nanjing Institute of Agricultural Mechanization, Ministry of Agriculture and Rural Affairs, Nanjing, China

**Keywords:** air-assisted nozzle, sprayer, droplet size, liquid flow rate, droplet coverage

## Abstract

This article reports the design and experiment of a novel air-assisted nozzle for pesticide application in orchard. A novel air-assisted nozzle was designed based on the transverse jet atomization pattern. This article conducted the performance and deposition experiments and established the mathematical model of volume median diameter (D_50_) and liquid flow rate with the nozzle design parameters. The D_50_ of this air-assisted nozzle ranged from 52.45 μm to 113.67 μm, and the liquid flow rate ranged from 142.6 ml/min to 1,607.8 ml/min within the designed conditions. These performances meet the low-volume and ultra-low-volume pesticide application in orchard. The droplet deposition experiment results demonstrated that the droplet coverage distribution in different layers and columns is relatively uniform, and the predicted value of spray penetration (*SP*) numbers *SP_iA_*, *SP_iB_*, and *SP_iC_* (*i* = 1, 2, and 3) are approximately 70%, 60%, and 70%, respectively. The droplet deposits on the foliage of the canopy (inside and outside) uniformly bring benefit for plant protection and pesticide saving. Compared with the traditional air-assisted nozzle that adopts a coaxial flow atomization pattern, the atomization efficiency of this air-assisted nozzle is higher. Moreover, the nozzle air pressure and liquid flow rate are considerably lower and greater than the traditional air-assisted nozzle, and these results proved that this air-assisted nozzle has great potential in orchard pesticide application. The relationship between the D_50_ and nozzle liquid pressure of this air-assisted nozzle differs from that of traditional air-assisted nozzles due to the atomization pattern and process. While this article provides an explanation for this relationship, further study about the atomization process and mechanism is needed so as to improve the performance.

## 1 Introduction

In the last decade, with the growth of the global fruit trade market, the high-density orchard model, which is suitable for mechanized management, has been widely promoted and adopted worldwide to continuously produce high-yielding and high-quality fruit production (Phuyal et al., 2020; Ou et al., 2024). Pest control is a crucial aspect of orchard management, which plays a significant role in ensuring the safety and quality of agricultural products (Zuoping et al., 2014; Appah et al., 2019). In high-density orchards, the use of pesticides has become essential for producing high-quality fruit. The nozzle is the key component for pesticide sprayers, and a conventional atomizing nozzle is inadequate for the fine atomization and high efficiency requirements of orchard pesticide application. Air-assisted sprayers guarantee that most of the fine pesticide droplets could deposited on the target surface, which were used to eradicate pests and prevent crop damage (Li et al., 2020a; Zhang et al., 2022). The liquid atomization process in the air-assisted nozzle depends on the collision and friction force resulting from the air–liquid velocity difference (Li et al., 2021; Wang et al., 2021). Nozzle atomization is a complex, multiphase, and transient process; it consumes a significant amount of atomization energy to break the liquid into liquid film or filament at the nozzle outlet. The liquid film or filament spreads into the break point by high-speed air, creating a large air–liquid velocity difference and finally forming droplets (Zhao et al., 2019). The air–liquid mass ratio had an effect on the nozzles. As the air–liquid mass ratio increases, the interaction between the air–liquid phase becomes stronger. The air–liquid mass ratio also has a great influence on the droplet size of the coaxial air–liquid atomization nozzle (Broumand et al., 2020; Chu et al., 2020). The air–liquid ratio can significantly improve the atomization effect of the nozzle and achieve fine atomization and low-capacity application; it can also reduce the droplet drift and improve the droplet deposition uniformity in the orchard pesticide application (Kang et al., 2018; Boiko et al., 2019; Chen et al., 2020). Some experimental studies were conducted for the mathematical model of the air-assisted nozzle’s atomization performance, and results indicated that the air in the nozzle has a significant influence on the droplet atomization quality (Czaczyk, 2012; Pizziol et al., 2017). The studies confirmed that the coaxial air–liquid air-assisted nozzle can effectively reduce the pesticide consumption and environmental pollution in the pesticide application (Patel et al., 2016, 2017). The similar study indicated that the number of liquid pores, liquid hole diameter, and stomatal diameter have different effects on the liquid flow rate and air rate performance of air-assisted atomization nozzles (Wang et al., 2019). An experimental study of fan-assisted nozzles revealed that the distribution uniformity of droplets firstly decreased and subsequently increased, and the droplet size firstly increased and subsequently decreased with the increased liquid pressure (Li et al., 2020b).

In this study, a novel air-assisted nozzle, comprising an air flow part and a nozzle cover, is designed. The inner chamber of the air flow part consists of a cylindrical section and a conical section, the liquid flowing into the high-velocity air along the radial direction around the nozzle outlet. The effects of nozzle structure and working parameters on droplet size and liquid flow rate were studied through laboratory experiments. The droplet deposition experiment was conducted to analyze the droplet coverage and penetration within the imitated tree canopy. The results provide valuable experiences for the development of high-performance air-assisted sprayers.

## 2 Materials and methods

### 2.1 Air-assisted nozzle design

**Figure 1:**
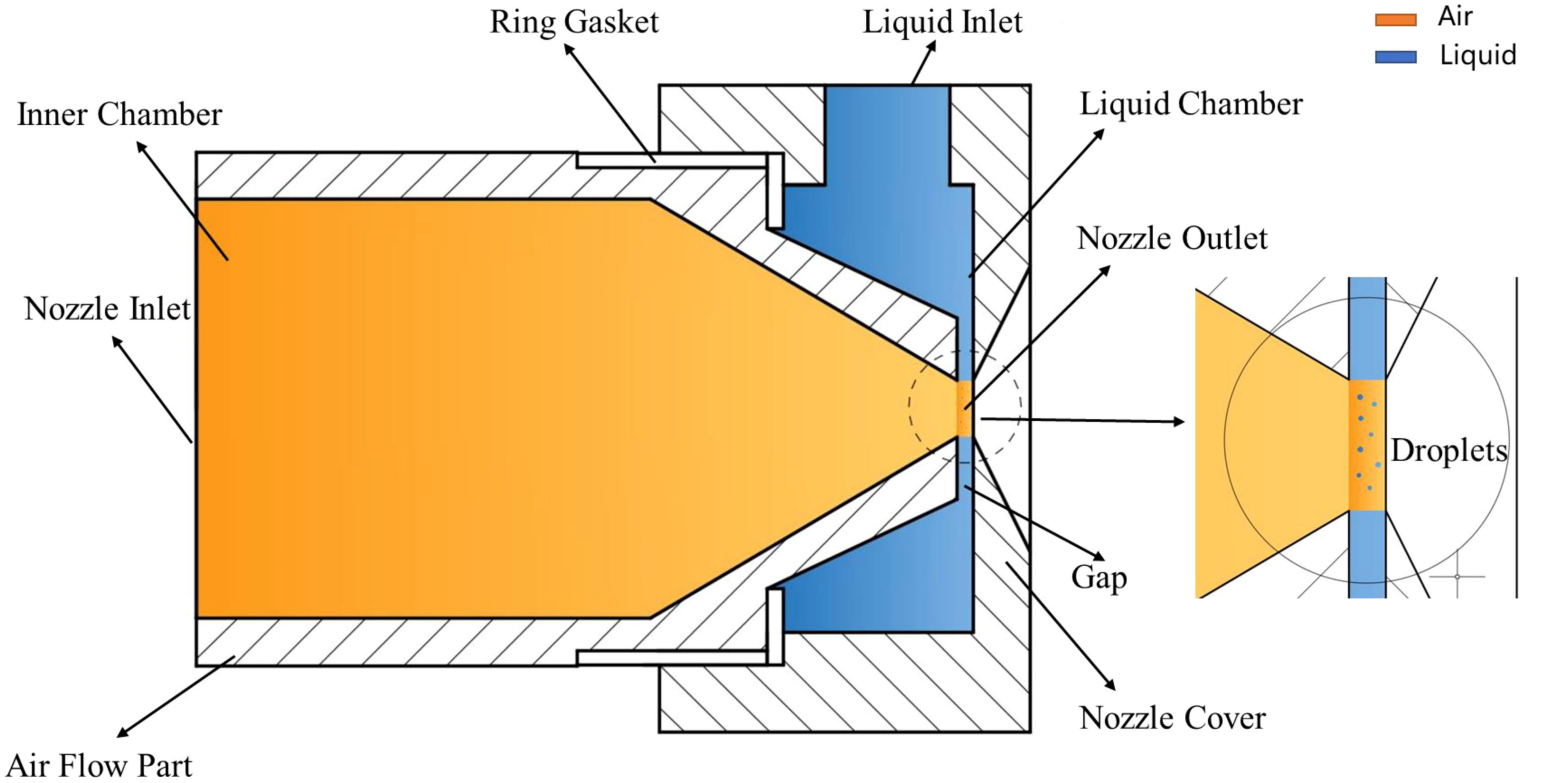
Structure of the air-assisted atomization nozzle.

The atomization process of air-assisted nozzles is a complex air–liquid interaction. High-velocity air flows improve the atomization effect of nozzles and disturb the canopy of fruit trees in the orchard pesticide application, so as to improve the deposition rate and reduce the usage of pesticide (Salcedo et al., 2019; Wang et al., 2022a). The air-assisted nozzle in this article is designed based on the transverse jet atomization pattern, and the nozzle consists of the air flow part and nozzle cover, as shown in **Figure 1**. The high-pressure air enters the air flow part from the nozzle inlet and forms high-velocity transverse airflow at the nozzle outlet, and the liquid is atomized into droplets by the transverse air flow at the nozzle outlet. The liquid jet during this atomization process is an annular jet form when it passes the gap between the air flow part and the nozzle cover; this annular jet form increases the air–liquid contact area significantly compared with the traditional cylindrical jet form. In order to reduce the flow losses inside the nozzle and improve the airflow velocity in the nozzle outlet, the inner chamber of the air flow part is divided into cylindrical section and conical section; this design minimizes the flow losses when the high-pressure air passes the nozzle and is suitable for multi-nozzle sprayer development. The liquid is delivered from the liquid inlet to the liquid chamber, which is located between the air flow part and nozzle cover. Then, it flows into the high-velocity transverse airflow along the radial direction through the gap which is located between the end of the air flow part and the nozzle cover. Subsequently, the liquid is atomized into droplets in the nozzle outlet. The structural diagram is shown in **Figure 1**. This nozzle has a compact structure and a high-efficiency flow pattern.

Based on the governing equation for frictionless, adiabatic, steady, and one-dimensional isentropic compressible flow, under certain pressure and temperature conditions, the airflow velocity increases when it passes through a confined space. The pressure at the nozzle inlet is fixed for nozzle design. Once the environment pressure decreases, it results in an increase in the mass flow rate. A “choking” phenomenon occurs when the airflow velocity in the nozzle outlet reaches the speed of local sound. At this point, the airflow velocity is related to the temperature and pressure at the nozzle inlet. The airflow velocity *V*, nozzle inlet pressure *P*_1_, and environment pressure *P*_2_ are calculated with Equations (1), (2) and as follows:


(1) $$\frac{P_{2}}{P_{1}}={\left(\frac{2}{\gamma +1}\right)}^{\frac{\gamma }{\gamma -1}}\;\;\;\;\;\;$$

(2) $$\;V=\sqrt{\frac{2\gamma }{\gamma -1}\times \frac{P_{2}}{\rho_{a}}\times \left[{\left(\frac{P_{1}}{P_{2}}\right)}^{\frac{\gamma -1}{\gamma }}-1\right]}$$

where *P*_1_ is the inlet pressure of the nozzle (MPa); *P*_2_ is the environment pressure (MPa); *γ* is the adiabatic exponent, as 1.4 in nozzle design; *V* is the airflow velocity in the confined space (m/s); ρ_a_ is the air density (kg/m); and V refers to the airflow velocity at the nozzle outlet. The nozzle inlet air flow can be calculated with Equation (3) as follows.


(3) $$Q_{in}=\frac{V\pi \emptyset_{1}^{2}}{4}$$

where *Q_in_* is the nozzle inlet air flow (m^3^/h) and ∅_1_ is the nozzle outlet diameter (mm).

The shape and size of the nozzle cover are determined according to the size of the air flow part. The nozzle outlet diameter ∅_1_ is designed for 6 mm, 8 mm, and 10 mm; the nozzle inlet diameter is 44 mm, and the shrinking angle of the conical section is 60°. The ring gasket is used to adjust the gap width, which was designed for 0.2 mm, 0.4 mm, and 0.8 mm.

### 2.2 Experiment design of droplet size measurement

#### 2.2.1 Experiment system design

**Figure 2:**
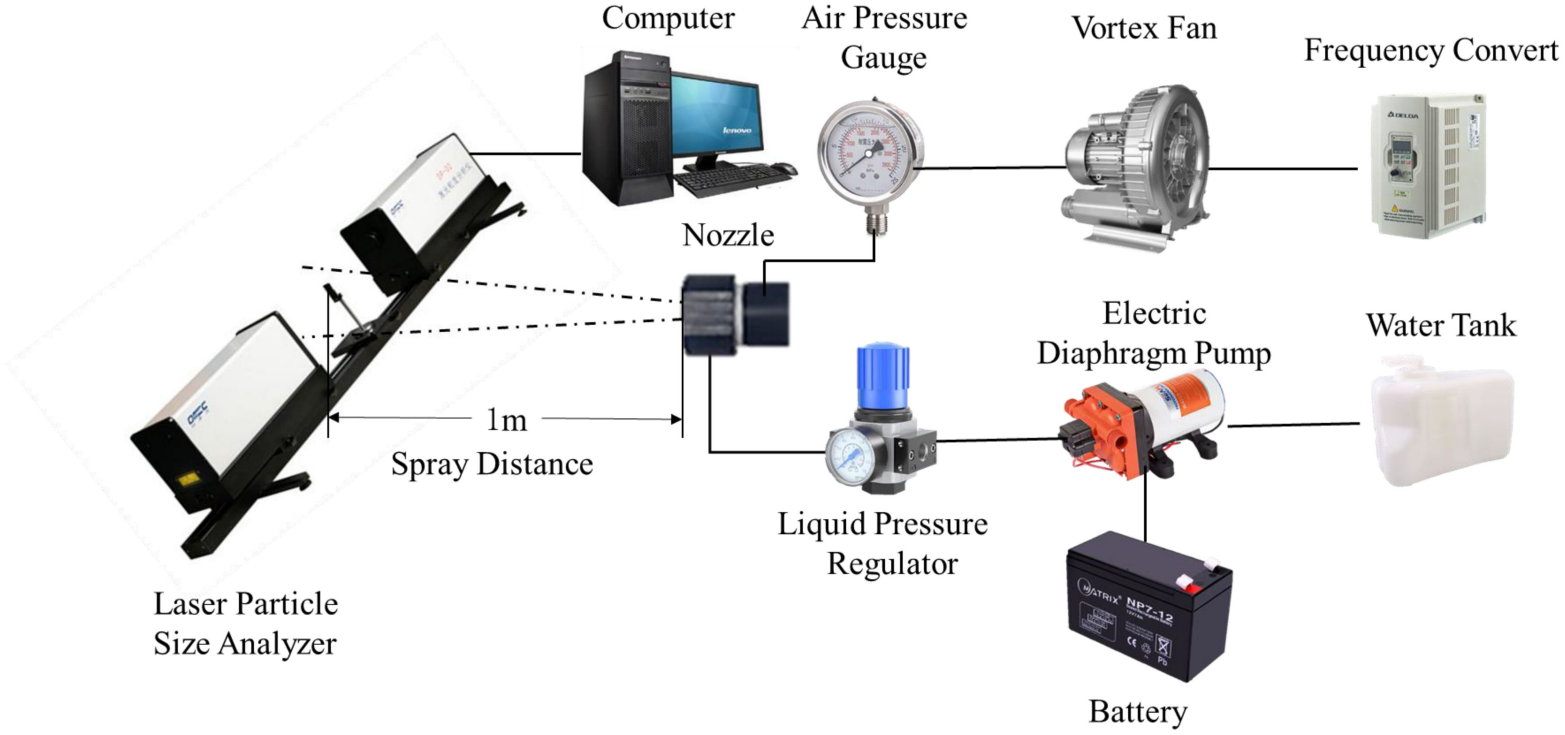
Experiment system of the droplet size measurement.

The droplet size is used to describe the atomization performance of the air-assisted nozzle, and it has a significant effect on the deposition distribution, drift, and deposition rate. The volume median diameter (D_50_) is used to characterize the atomization performance in this article (Nogueira Martins et al., 2021; Yu et al., 2021). The experiment system to measure the droplet size included the air-supplied spray subsystem and the droplet size measurement subsystem, as shown in **Figure 2**. The air-supplied spray subsystem included vortex fan, frequency converter, air pressure gauge, air-assisted nozzle, water tank, electric diaphragm pump, battery, and liquid pressure regulator valve. The vortex fan (ASBA HG-2200S) provided high-pressure air flows for the air-assisted nozzle. The frequency converter (model: Instar) adjusted the air flows by changing the rotational speed of the vortex fan, and the air pressure in the nozzle inlet was measured by the air pressure gauge. The electric diaphragm pump (SEAFLO SFDP2) is powered by battery and delivers the liquid (tap water) from the water tank into the air-assisted nozzle. The liquid pressure regulator is used to adjust the liquid flow rate of the air-assisted nozzle. The droplet size measurement subsystem included the laser particle size analyzer (OMEC DP-02) and computer, and it was used to measure the droplet size information of nozzle in indoor test condition (Dai et al., 2022). The air-assisted nozzle used in the experiment was made by Teflon and manufactured by a CNC (computer numerical control) machine; the surface of the nozzle was very smooth and beneficial to reduce the flow losses, the tap water was very clean, and there was no filter before the liquid inlet during the experiments. The air-assisted nozzle was fixed on the test bench, the spray distance was set to 0.8 m according to the nozzle performance pre-test and previous experiences, and the air-assisted nozzle and laser beam were set in the same horizontal plane to ensure that the droplets could be fully collected by the laser particle size analyzer. Each experiment was repeated three times, and the mean value was taken as the experiment result in the analysis.

#### 2.2.2 Experiment parameter design

The air–liquid velocity difference is an important factor affecting the spray nozzle atomization effect. The nozzle outlet diameter and air pressure in the nozzle inlet (nozzle air pressure) are significant factors affecting the airflow velocity in the nozzle outlet. The gap width and liquid pressure in the liquid inlet (nozzle liquid pressure) are significant factors affecting the liquid velocity in the atomization (Ishimoto et al., 2008). The hybrid horizontal orthogonal experiment is conducted in this article. The nozzle outlet diameter *d*, gap width *w*, nozzle air pressure P*_g_*, and nozzle liquid pressure *P_l_* were variables in the experiment, and a mathematical model of D_50_ based on these variables was established (Miranda-Fuentes et al., 2018). The variable level table and experiment design are shown in **Tables 1** and **2**.

**Table 1:** Variable level table of droplet size measurement experiment.

*Level*	*A*	*B*	*C*	*D*
	*Outlet diameter (mm)*	*Gap width (mm)*	*Nozzle air pressure (*MPa*)*	*Nozzle liquid pressure (*MPa*)*
1	6	0.2	0.010	0.02
2	8	0.4	0.015	0.03
3	10	0.6	0.020	0.04
4	—	—	—	0.05
5	—	—	—	0.06

**Table 2:** Orthogonal design scheme of droplet size measurement.

*Number*	*Factor*			
	*A*	*B*	*C*	*D*
1	1	1	1	1
2	1	1	2	2
3	1	2	1	3
4	1	2	3	4
5	1	3	2	5
6	1	3	3	1
7	2	1	1	1
8	2	1	3	5
9	2	2	2	1
10	2	2	3	2
11	2	3	1	4
12	2	3	2	3
13	3	1	2	4
14	3	1	3	3
15	3	2	1	5
16	3	2	2	1
17	3	3	1	2
18	3	3	3	1

The environment temperature was 20°C ± 2°C, and the ambient humidity was 38% ± 5%.

### 2.3 Experiment design of liquid flow rate measurement

#### 2.3.1 Experiment system design

**Figure 3:**
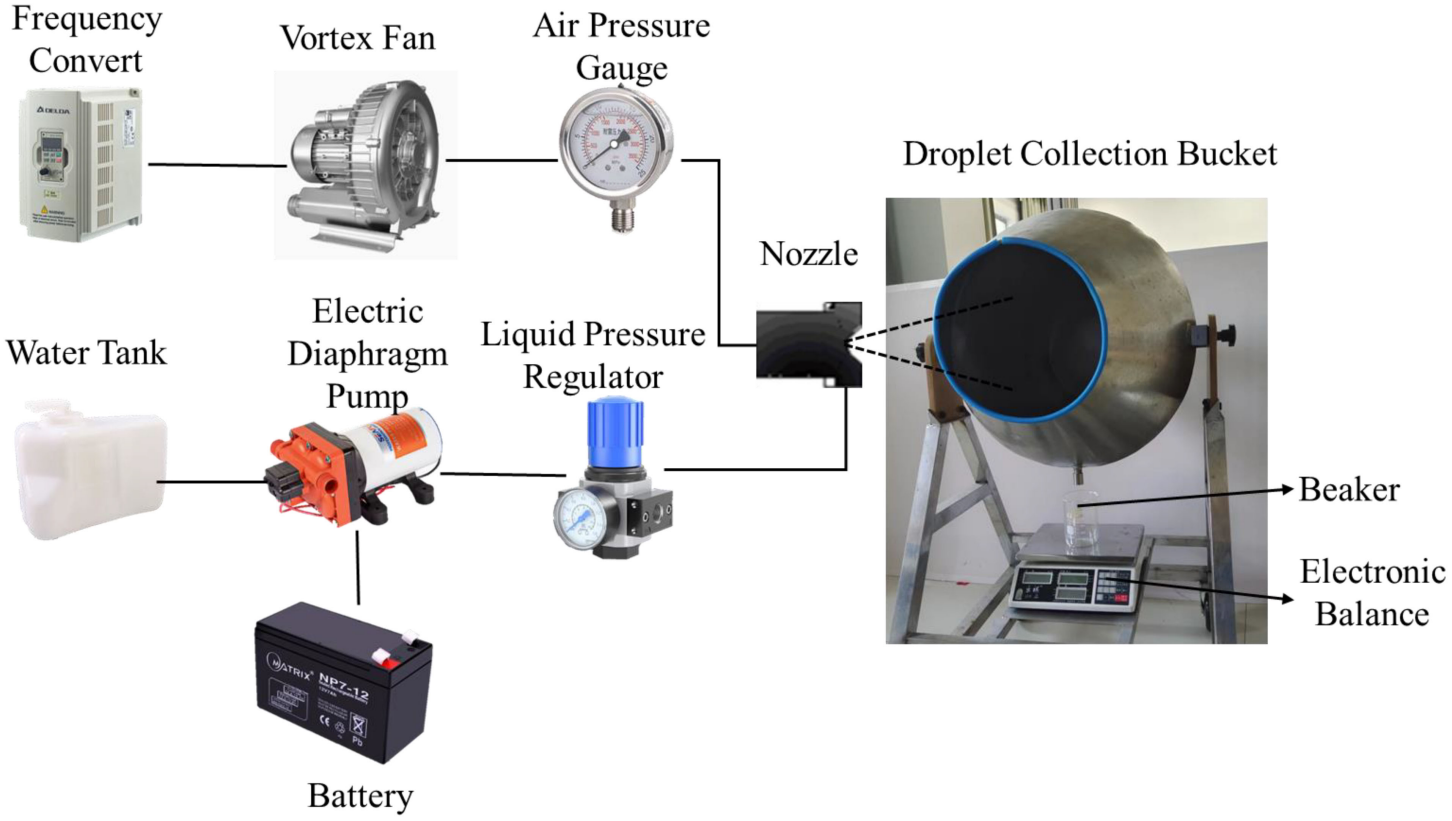
Experiment system of the liquid flow rate measurement.

Liquid flow rate is one of the key parameters for the air-assisted nozzle. A weighing method was used for liquid flow rate measurement in this study. The liquid flow rate measurement system included an air-supplied spray subsystem and a liquid flow rate measurement subsystem, as shown in **Figure 3**. The air-supplied spray subsystem was stated before, and the liquid flow rate measurement subsystem included a droplet collecting device and an electronic balance (ACS-LQ300001). The droplet collection device included the droplet collection bucket and beaker. The droplet collection bucket was used to collect the droplets from the air-assisted nozzle, and the beaker was used to measure the droplets from the droplet collection bucket. After 1-min steady operation of experiment system, all the droplets have been collected in the beaker. Then, the mass of the beaker with liquid was measured by the electronic balance, and the liquid flow rate was calculated using the results. Each group of experiments was repeated three times, and the average value of the three experiments was taken as the experiment result.

#### 2.3.2 Experiment parameter design

The hybrid horizontal orthogonal experiment was designed for air-assisted nozzle performance research. The nozzle outlet diameter *d*, gap width *w*, and nozzle liquid pressure *P_l_* were variables in the experiment. The nozzle air pressure *P_g_* was set to 0.02 MPa, and the mathematical model of liquid flow rate based on these variables was established (Miranda-Fuentes et al., 2015). The variable level table and experiment design are shown in **Tables 3** and **4**, respectively.

**Table 3:** Variable level table of liquid flow measurement experiment.

*Level*	*A*	*B*	*D*
	*Nozzle outlet diameter (mm)*	*Gap width (mm)*	*Nozzle liquid pressure (*MPa*)*
1	6	0.2	0.02
2	8	0.4	0.03
3	10	0.6	0.04
4	—	—	0.05
5	—	—	0.06

**Table 4:** Orthogonal experiment scheme of liquid flow rate measurement.

*Number*	*Factor*		
	*A*	*B*	*D*
1	1	1	1
2	1	1	2
3	1	2	3
4	1	2	4
5	1	3	5
6	1	3	1
7	2	1	1
8	2	1	5
9	2	2	1
10	2	2	2
11	2	3	4
12	2	3	3
13	3	1	4
14	3	1	3
15	3	2	5
16	3	2	1
17	3	3	2
18	3	3	1

The environment temperature was 20°C ± 2°C, and the ambient humidity was 38% ± 5%.

### 2.4 Experiment design of droplet deposition

#### 2.4.1 Experiment system design

**Figure 4:**
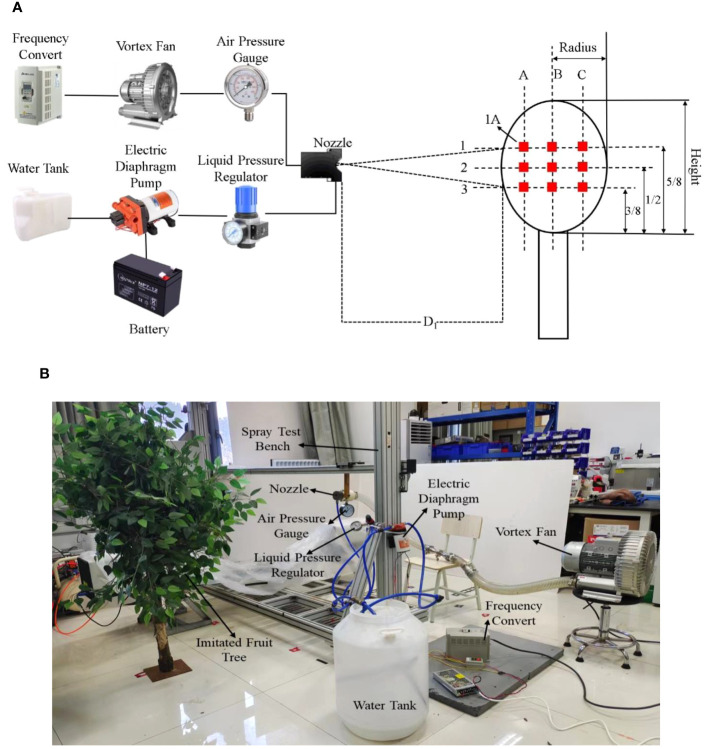
Droplet deposition experimental system. **(A)** is the schematic diagram of the droplet deposition experiment system; **(B)** is the experiment system site picture.

In order to study the droplet deposition in the tree canopy, the imitated tree canopy was selected in the droplet deposition experiment. The height and radius of the tree were approximately 1.0 m and 0.5 m, respectively, and the average LAI (leaf area index) of the imitated tree canopy was approximately 5.9. The water-sensitive paper (26 × 76 mm) was used to measure the droplet coverage and deposition density, which were used to assess the deposition effect of the air-assisted nozzle (Nishida et al., 2012; Ventura et al., 2018). Nine water-sensitive paper layout points were set at different locations in the imitated tree canopy, and the imitated tree canopy was divided into three layers (1, 2, 3) and columns (A,B,C) in the vertical and horizontal directions (Wang et al., 2022b). The water-sensitive paper layout points were numbered according to the layer and column, as shown in **Figure 4A**.

The deposition experiment system included the air-supplied spray subsystem and imitated tree canopy, as shown in **Figure 4B**. The nozzle was fixed on the spray test bench, which provided a spaying speed of 0 m/s~1 m/s; the spraying speed was 1 m/s; the spraying distance (D_1_) was 0.8 m; and the air-assisted nozzle was located at the height of layer 2 in the experiment (Dai et al., 2023). According to the results of nozzle droplet size and flow measurement, the nozzle outlet diameter and gap width were 6 mm and 0.4 mm, respectively, and the air pressure and liquid pressure were 0.02 MPa and 0.05 MPa respectively in the droplet deposition experiment. The environment temperature was 20°C ± 2°C, and the ambient humidity was 38% ± 5%.

#### 2.4.2 Experiment data process method

Each group of experiments was repeated for three times, and the average value of the three experiments was taken as the droplet deposition experiment result. The water-sensitive papers after spraying operation were scanned with the scanner (M7628DNA, LENOVO) to obtain 8-bit greyscale images (600 dpi); the water-sensitive paper images were processed using “Deposit Scan” software to calculate the droplet coverage results (Zhu et al., 2011). The droplet coverage results in this experiment indicated the droplet deposition inside the imitated tree canopy, which is important to control the pests and diseases in orchard management; these results will also provide useful performance assessment for orchard pesticide sprayer development.

According to the water-sensitive paper layout, *C_ij_* represents the droplet coverage of water-sensitive paper which is located in layer *i* and column *j*, as shown in **Figure 4A**. In order to explore the droplet deposition inside the tree canopy, droplet coverage rate *C_i_* is defined as the sum of the *C_ij_* (*j*=A, B, C) and calculated according to Equation (4).


(4) $$C_{i}=C_{iA}+C_{iB}+\;C_{iC}$$

In order to assess the nozzle performance and droplet-air penetration effect into the tree canopy, spray penetration (*SP*) was defined as the ratio of *C_ij_* to *C_i_* and calculated according to Equation (5); this result is used for evaluating the penetration effect of droplet deposition inside the imitated tree canopy (Li et al., 2022).


(5) $$SP_{ij}=\frac{C_{ij}}{C_{i}}100\%$$

## 3 Results and discussion

### 3.1 Experiment result of droplet size measurement

The droplet size measurement result and variance analysis are shown in **Tables 5** and **6**, respectively. The result demonstrated that the D_50_ value was between 52.45 μm and 113.67 μm in the experiment.

**Table 5:** Experiment result of droplet size measurement.

*Number*	*Factor*				D_50_ *(μm)*
	*A*	*B*	*C*	*D*	
1	1	1	1	1	83.92
2	1	1	2	2	71.64
3	1	2	1	3	77.91
4	1	2	3	4	52.45
5	1	3	2	5	69.07
6	1	3	3	1	64.21
7	2	1	1	1	105.53
8	2	1	3	5	67.13
9	2	2	2	1	88.44
10	2	2	3	2	61.21
11	2	3	1	4	79.06
12	2	3	2	3	78.97
13	3	1	2	4	69.89
14	3	1	3	3	68.17
15	3	2	1	5	103.86
16	3	2	2	1	97.5
17	3	3	1	2	113.67
18	3	3	3	1	77.75
K1	419.20	466.28	563.95	517.35	—
K2	480.34	481.37	475.51	246.52	—
K3	530.84	482.73	390.92	225.05	—
K4				201.40	—
K5				240.06	—
k1	69.87	77.71	93.99	86.23	—
k2	80.06	80.23	79.25	82.17	—
k3	88.47	80.46	65.15	75.02	—
k4				67.13	—
k5				80.02	—
R	18.61	2.74	28.84	19.09	—

**Table 6:** Variance analysis of droplet size measurement experiment.

*Source*	*SS*	*df*	*MS*	*F*	*p*
*Corrected model*	4,377.604a	10	437.760	9.153	0.004
*Intercept*	101,695.749	1	101,695.749	2,126.429	0.000
*Outlet diameter*	1,041.769	2	520.884	10.892	0.007
*Gap width*	27.787	2	13.893	0.291	0.756
*Air pressure*	2,495.360	2	1,247.680	26.089	0.001
*Liquid pressure*	812.688	4	203.172	4.248	0.047
*Error*	334.773	7	47.825	—	—
*Total*	118,378.318	18	—	—	—
*Corrected total*	4,712.376	17	—	—	—

R^2 ^= 0.929 (revised R^2^ = 0.827).

**Figure 5:**
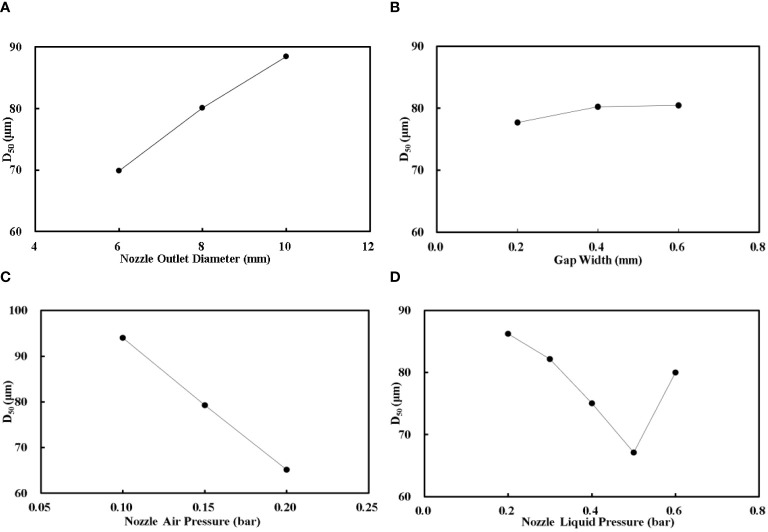
The variation trends of D_50_. **(A)** is the relationship between the nozzle outlet diameter with D_50_; **(B)** is the relationship between the gap width with D_50_; **(C)** is the relationship between the nozzle air pressure with D_50_; **(D)** is the relationship between the nozzle liquid pressure with D_50_.

The variation trends of D_50_ with the nozzle outlet diameter, gap width, nozzle air pressure, and nozzle liquid pressure are shown in **Figures 5A–D**, respectively. D_50_ increased with the increase of the nozzle outlet diameter and decreased with the increase of the nozzle air pressure. The gap width has no significant correlation with D_50_. With the increase of liquid pressure, D_50_ decreased firstly and increased afterward; there is a minimum value of D_50_ during the experiments, which is useful for the air-assisted nozzle design.

The nozzle outlet diameter, gap width, nozzle air pressure, and nozzle liquid pressure are the most important factors in nozzle development. As the nozzle outlet diameter increased, the airflow velocity in the nozzle outlet decreased, the air–liquid velocity difference decreased, and the interaction intensity between the air and liquid phase decreased accordingly, which caused the droplet size increase (Musiu et al., 2019). The gap width was adjusted by the ring gasket with different thicknesses during the experiment; once the gap width increased, the liquid flow rate increased accordingly, and then the atomization effect was reduced and the droplet size became larger (Jadhav and Deivanathan, 2020). With the increase of nozzle air pressure, the airflow velocity in the nozzle outlet increased, and the air–liquid velocity difference increased. The interaction intensity between the air and liquid phase increased accordingly, and then the droplet size became smaller (Balsari et al., 2019). As the nozzle liquid pressure increased, D_50_ decreased firstly and increased afterward. The increase of liquid velocity affects the mechanism of the liquid jet in the atomization space; the liquid flows into the nozzle outlet from the annular gap and forms an annular jet along the radial direction. This novel design is different from the conventional air-assisted nozzle used in pesticide application. The atomization performance of this design is also different with the conventional air-assisted nozzle; the nozzle air pressure of this novel air-assisted nozzle is lower and the liquid flow rate is greater than the conventional air-assisted nozzle (Amighi and Ashgriz, 2019; Han et al., 2020).

Linear regression analysis was conducted based on the droplet size measurement results, and the mathematical model of D_50_ was established as shown in Equation (6) (Liao et al., 2019). The linear regression analysis results of D_50_ are shown in **Table 7**.

**Table 7:** Linear regression analysis results of D_50_ measurement experiment.

	*Non-standard coefficient*		*Standard coefficient*	*t*	*p*	*Collinearity diagnosis*	
	*B*	*SE*	*Beta*			*VIF*	*Tolerance*
*Constant*	94.035	12.969	—	7.251	0.000	—	—
*Outlet diameter*	4.652	1.117	0.469	4.164	0.001	1.000	1.000
*Gap width*	6.854	11.170	0.069	0.614	0.550	1.000	1.000
*Air pressure*	−288.383	44.681	−0.728	−6.454	0.000	1.000	1.000
*Liquid pressure*	−30.727	12.236	−.283	−2.511	0.026	1.000	1.000
*R 2*	0.835						
*Revised R 2*	0.784						
*F*	F (4,13) = 16.420, p = 0.000						


(6) $${\text{D}}_{50}=94.035+4.652d+6.854w-28.838P_{g}-3.073P_{l}$$

where D_50_ is the volume median diameter (μm); *d* is the nozzle outlet diameter (mm); *w* is the gap width (mm); *P_g_* is the nozzle air pressure (MPa); and *P_l_* is the nozzle liquid pressure (MPa).

The data of **Table 7** show that the R^2^ value of the regression equation is 0.835 and the Revised R^2^ value is 0.784. The linear relationship between D_50_ and *d*, *w*, *P_g_*, and *P_l_* is significant, and there is a positive correlation between the nozzle outlet diameter and D_50_. Both the nozzle air pressure and nozzle liquid pressure have significant negative correlations with D_50_, and these two parameters are the most important factors during the nozzle design. The regression coefficient of gap width is 6.845(t = 0.614, p = 0.550 > 0.05), indicating that gap width has a smaller effect on D_50_. These results are basically consistent with the results of variance analysis, and the mathematical model based on the nozzle outlet diameter, gap width, nozzle air pressure, and nozzle liquid pressure is useful for the development of this novel air-assisted nozzle.

### 3.2 Experimental result of the liquid flow rate

The liquid flow rate measurement result and variance analysis are shown in **Tables 8** and **9**, respectively. The result demonstrated that the liquid flow rate was between 142.6 ml/min and 1607.8 ml/min in the experiment.

**Table 8:** Experimental results of liquid flow rate measurement.

*Number*	*Factor*			*Flow rate (ml/min)*
	*A*	*B*	*D*	
*1*	1	1	1	142.6
*2*	1	1	2	321.6
*3*	1	2	3	559.3
*4*	1	2	4	734.3
*5*	1	3	5	1,007.1
*6*	1	3	1	493.5
*7*	2	1	1	187.8
*8*	2	1	5	642.3
*9*	2	2	1	380.9
*10*	2	2	2	695.5
*11*	2	3	4	817.2
*12*	2	3	3	630.9
*13*	3	1	4	645.6
*14*	3	1	3	541.5
*15*	3	2	5	1,607.8
*16*	3	2	1	689.1
*17*	3	3	2	1,041.8
*18*	3	3	1	695.4
*K1*	3,258.4	2,481.4	2,589.3	—
*K2*	3,354.6	4,666.9	1,731.7	—
*K3*	5,221.2	4,685.9	2,058.9	—
*K4*			2,197.1	—
*K5*			3,257.2	—
*k1*	543.1	413.6	431.6	—
*k2*	559.1	777.8	577.2	
*k3*	870.2	781.0	686.3	
*k4*			732.4	—
*k5*			1,085.7	—
*R*	327.1	367.4	654.2	—

**Table 9:** Variance analysis table of liquid flow rate measurement experiment.

*Source*	*SS*	*df*	*MS*	*F*	*p*
*Corrected model*	1,838,582.515a	8	229,822.814	21.585	0.000
*Intercept*	8,228,304.756	1	8,228,304.756	772.793	0.000
*Outlet diameter*	408,112.991	2	204,056.496	19.165	0.001
*Gap width*	535,366.194	2	267,683.097	25.140	0.000
*Liquid pressure*	895,103.329	4	223,775.832	21.017	0.000
*Error*	95,827.409	9	10,647.490	—	—
*Total*	9,714,870.460	18	—	—	—
*Corrected total*	1,934,409.924	17	—	—	—

R^2 ^= 0.950(Revised R^2^ = 0.906).

**Figure 6:**
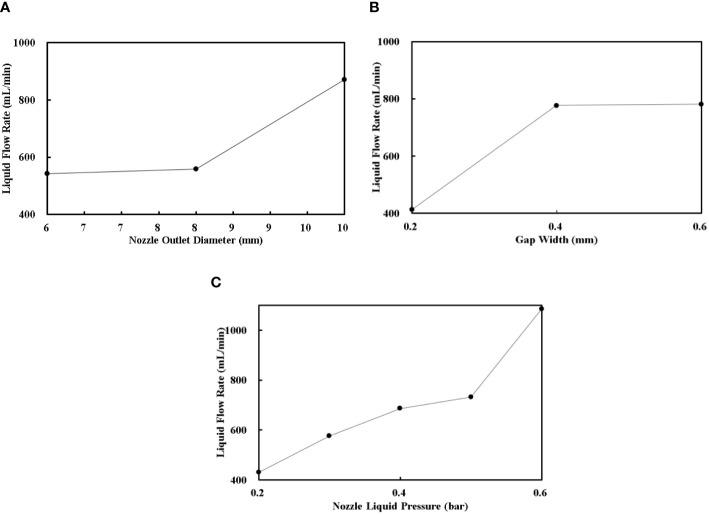
The variation trends of the liquid flow rate. **(A)** is the relationship between the nozzle outlet diameter with the liquid flow rate; **(B)** is the relationship between the gap width with the liquid flow rate; **(C)** is the relationship between the nozzle liquid pressure with the liquid flow rate.

The variation trends of the liquid flow rate with the nozzle outlet diameter, gap width, and nozzle liquid pressure are shown in **Figures 6A–C**, respectively. The liquid flow rate increased with the increase of the nozzle outlet diameter, and it increased firstly and remained unchanged afterward with increase of the gap width. Meanwhile, the liquid flow rate increased with the increase of the nozzle liquid pressure gradually.

The relationship between the liquid flow rate and the nozzle outlet diameter, gap width, and nozzle liquid pressure are governed by fluid resistance and friction loss theory. The nozzle outlet diameter and gap width determine the flow cross section for this air-assisted nozzle, and the nozzle liquid pressure provides the original fluid dynamic energy for the liquid (Wang et al., 2020; Xue et al., 2021).

Linear regression analysis was conducted based the liquid flow rate measurement results; the mathematical model of *Q* was established as shown in Equation (7). The linear regression analysis results of *Q* were shown in **Table 10**.

**Table 10:** Linear regression analysis results of liquid flow measurement experiment.

	*Non-standard coefficient*		*Standard coefficient*	*t*	*p*	*Collinearity diagnosis*	
	*B*	*SE*	*Beta*			*VIF*	*Tolerance*
*Constant*	−890.938	237.292		−3.755	0.002	—	—
*Outlet diameter*	81.783	23.873	0.407	3.426	0.004	1.000	1.000
*Gap width*	918.542	238.729	0.458	3.848	0.002	1.000	1.000
*Liquid pressure*	1,436.483	261.514	0.653	5.493	0.000	1.000	1.000
*R2*	0.802						
*Revised R2*	0.760						
*F*	F(3,14)= 18.904, p = 0.000						


(7) $$Q=81.783d+918.542w+143.648P_{l}–890.938$$

where *Q* is the liquid flow rate (ml/min); *d* is the nozzle outlet diameter (mm); *w* is the gap width (mm); and *P_l_* is the nozzle liquid pressure (MPa).

The data of **Table 10** show that the R^2^ value of the regression equation is 0.802 and the adjusted R^2^ value is 0.760. The linear relationship between *Q* and *d*, *w*, and *P_l_* is significant, and the nozzle outlet diameter, gap width, and nozzle liquid pressure all have significant positive correlations with the liquid flow rate. These results are consistent with the fluid resistance and friction loss, and mathematical model is useful for the development of this novel air-assisted nozzle.

### 3.3 Experimental result of the droplet deposition

According to the above experiment results and requirement of the pesticide application in orchard, the parameters of the air-assisted nozzle used for droplet deposition experiment are as a flower: the nozzle outlet diameter is 6 mm; the gap width is 0.4 mm; the nozzle air pressure is 0.02 MPa; and the nozzle liquid pressure is 0.05 MPa.

**Figure 7:**
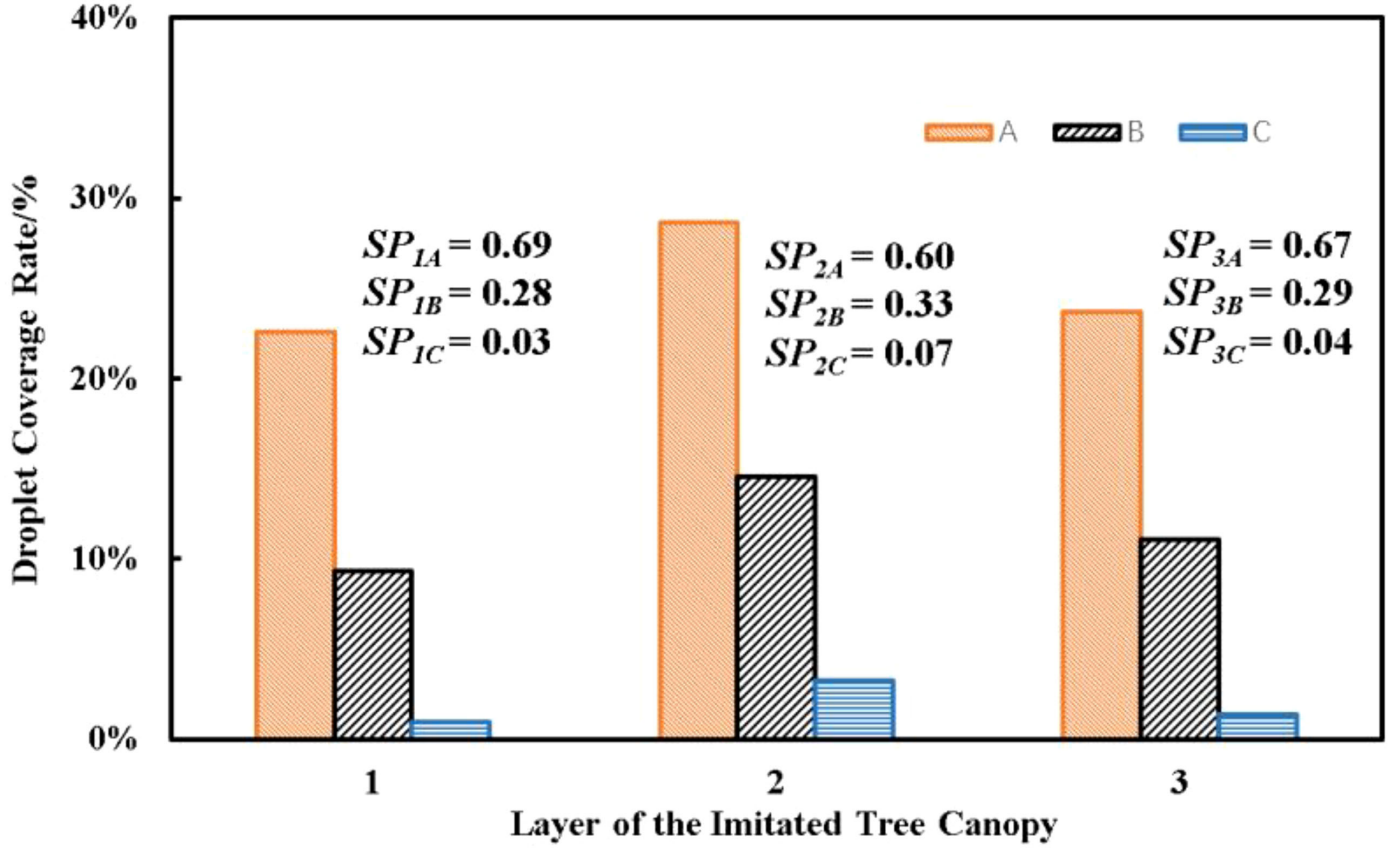
The results of droplet coverage and spray penetration.

The droplet deposition experiment was conducted for valuing the droplet deposition results in the imitated tree canopy condition. The results of droplet deposition experiment are shown in **Figure 7**. With the distance between the air-assisted nozzle and the water-sensitive paper increasing, the droplet coverage decreased significantly in all the three layers, and C*_iA_* > C*_iB_* > C*_iC_* (*i* = 1, 2, and 3) is clearly observed from the results. The results demonstrated that *SP_1A_*, *SP_2A_*, and *SP_3A_* are approximately 69%, 60%, and 67%, respectively; *SP_1B_*, *SP_2B_*, and *SP_3B_* are approximately 28%, 33%, and 29%, and the *SP_1C_*, *SP_2C_*, and *SP_3C_* are approximately 3%, 7%, and 4%, respectively. The average droplet coverage of water-sensitive paper in column A is approximately 65% of the total droplet coverage; meanwhile, there are approximately 30% and 5% of average droplet coverage in columns B and C, respectively.

The droplet coverage decreased due to the increase of spray distance and canopy obstacle effect. These data indicated that most of the droplet–air jet from the air-assisted nozzle reached columns A and B, and the average values of droplet coverage in columns A, B, and C are approximately 25%, 12%, and 2%, respectively. The pesticide sprayer normally sprays one row twice and operates from the two sides of the tree, respectively; according to the operation method in orchard, the predicted values of *SP_iA_*, *SP_iB_*, and *SP_iC_* (*i* = 1, 2, and 3) are approximately 70%, 60%, and 70%, and the predicted values of droplet coverage in columns A, B, and C are approximately 27%, 24%, and 27%, respectively. These results indicated that the *SP* results in the columns are good and meet the pesticide penetration requirement in the orchard; the droplet coverage distribution in different columns is uniform and could be adjusted in the actual pesticide application according the pesticide requirement (Duga et al., 2015; Ferguson et al., 2016).

Within the same column, the droplet coverage values of different layers are close to each other. The maximum of droplet coverage differences in C*_iA_*, C*_iB_*, and C*_iC_* (*i* = 1, 2, and 3) are approximately 4%, 3%, and 1%, respectively. The droplet coverage in layer 2 is greater than in layer 1 and 3 because the nozzle centerline and layer 2 are in the same height, and both the droplet and air velocity near the nozzle centerline are greater than in other spaces. The droplet coverage in layer 3 is slightly greater than in layer 1 due to the gravity effect. These results indicate that the droplet deposition is significantly affected by the inertia and air jet. Furthermore, the droplet and air jet distribution of the air-assisted nozzle are uniform for orchard pesticide application.

## 4 Conclusions

This article provided the design and experiment studies of a novel air-assisted nozzle used in orchard pesticide application. This study established the mathematical model of volume median diameter and liquid flow rate with the design parameters and provided useful prediction models for nozzle development. The droplet deposition experiment was conducted using an imitated tree canopy for droplet deposition evaluation in realistic orchard condition, and the results proved that this air-assisted nozzle has good droplet coverage for pesticide application. According to the pesticide application method in orchard, the predicted droplet coverage distribution in different layers and columns are relatively uniform and the droplet deposition in the inside canopy is basically equal with the outside canopy. This is beneficial for plant protection and pesticide precision spraying.

The experiment results demonstrated that this air-assisted nozzle has advantages in nozzle air pressure and droplet atomization performance, and the volume median diameter and flow rate of this nozzle are 52.45 μm and 734.3 ml/min, respectively; under the design conditions, these performances are suitable for orchard sprayer development, which could meet the low-volume and ultra-low volume pesticide application requirements. The air pressure of this air-assisted nozzle is only approximately one-fourth of the inlet air pressure of MaxCharge nozzle development by Electrostatic Spray System Company (Pascuzzi and Cerruto, 2015), the D_50_ of these two nozzles are about the same, and the flow rate is approximately three times that of the MaxCharge nozzle; these parameters are important for orchard sprayer development and increasing the operation efficiency. These results reveal that the structure design of this air-assisted nozzle enhanced the atomization ability. Specifically, this air-assisted nozzle uses the transverse jet atomization pattern in the design instead of the coaxial flow atomization pattern, which is used widely in a traditional air-assisted nozzle, and the atomization efficiency of this air-assisted nozzle is higher than the traditional air-assisted nozzle. In addition, the traditional air-assisted nozzle normally uses liquid tubular jet flow into high-velocity airflow for atomization. The liquid in this air-assisted nozzle flows into the atomization space (nozzle outlet) along the radial direction as annular jet instead of the tubular jet, and the cross section of annular jet flow is bigger than the tubular jet and brings a greater liquid flow rate to this air-assisted nozzle. Therefore, the sprayer uses this air-assisted nozzle, and this advantage is beneficial for sprayer design and pesticide application.

The droplet size experiment results demonstrated that there is minimum value of volume median diameter when the nozzle liquid pressure increased. Nevertheless, the volume median diameter has a negative relationship with the nozzle liquid pressure in the nozzle using the coaxial flow atomization pattern. It can be inferred that when the annular jet flows into the center of the nozzle outlet with the increase of the nozzle liquid pressure, once the annular jet reaches the center of the nozzle outlet, the air–liquid velocity difference reaches the maximum, and the volume median diameter reaches the minimum. When the nozzle liquid pressure increases continuedly, the liquid flows outward after reaching the center of the nozzle outlet and the air–liquid velocity difference starts to decrease after the maximum. Although some basic theory and experiment results are completed in this study, the atomization mechanism of this air-assisted nozzle using typical pesticide is not clear, and the droplet deposition experiment results using imitated tree only proved the preliminary deposition performance and applicability. Considering that the pesticide has influence on the atomization mechanism and the droplet deposition is usually influenced by leaf size, LAI, spaying distance, spaying speed, working parameters, and environmental wind, the nozzle atomization mechanism and deposition performance are very complex issues under a real-world operating environment; further studies about the atomization and droplet deposition in real-world are needed and contribute to the application.

## Data availability statement

The original contributions presented in the study are included in the article/supplementary material. Further inquiries can be directed to the corresponding authors.

## Author contributions

MO: Conceptualization, Data curation, Formal analysis, Supervision, Writing – review & editing. JZ: Data curation, Formal analysis, Investigation, Validation, Writing – original draft, Writing – review & editing. WD: Formal analysis, Investigation, Writing – original draft. MW: Formal analysis, Investigation, Writing – original draft. TG: Data curation, Formal analysis, Investigation, Writing – original draft. WJ: Conceptualization, Methodology, Writing – review & editing. XD: Conceptualization, Data curation, Supervision, Writing – review & editing. TZ: Conceptualization, Formal analysis, Writing – review & editing. SD: Conceptualization, Data curation, Writing – review & editing.
